# Response to the Commentary from A Dissolution Working Group on “The Effect of Sampling Cannula on *In Vitro* Dissolution Testing with USP Paddle Method”: Clarifications and Opportunities for Engagement

**DOI:** 10.1208/s12248-025-01107-9

**Published:** 2025-07-16

**Authors:** Zongming Gao, Anjanette Smith

**Affiliations:** 1 Food and Drug Administration, Center for Drug Evaluation and Research, Division of Pharmaceutical Quality Research II, Office of Pharmaceutical Quality Research, St. Louis, Missouri 63110, United States of America; 2 Food and Drug Administration, Center for Drug Evaluation and Research, Division of Pharmaceutical Quality Research I, Office of Pharmaceutical Quality Research, St. Louis, Missouri 63110, United States of America

In April 2023, our paper entitled “The Effect of Sampling Cannula on *In Vitro* Dissolution Testing with USP Paddle Method” was published in AAPS Journal. This study examined the impact of sampling cannula size and setting on *in vitro* dissolution test of prednisone disintegrating tablets. The study concluded that both the size and setting of the sampling cannula may introduce significant systematic errors, even when the dissolution apparatus has been calibrated. The degree of interference in dissolution results was found to be directly related to the outer diameter of the sampling cannula. Consequently, the study recommended that the size of the sampling cannula and the sampling procedure should be documented in standard operating procedures (SOP) for dissolution testing during method development (1).

This publication garnered attention from members of the International Consortium for Innovation & Quality (IQ) dissolution working group (DWG). In April 2024, Abend et al., representing the IQ’s DWG, published a commentary in AAPS Journal addressing the aforementioned study (2). The commentary acknowledged, as stated in the original publication, that sampling cannula size, sampling procedure, and other factors may influence the results of *in vitro* dissolution tests. The commentary further noted that if sampling technique and cannula size are critical for a particular drug product, they should be specified in the dissolution procedure, which is part of the product specification. However, the IQ’s DWG expressed concern that “the conclusions in the FDA publication could raise expectations that experimental conditions, which are described in the dissolution procedure, must specify cannula size and sampling procedure even when the dissolution results are not affected by these.”

We welcome comments on our published research and share interests with scientists in the field, as this dialogue can contribute to a better understanding of factors influencing *in vitro* dissolution testing. It is recognized that variations in dissolution results may stem from various sources, including the lack of robustness in the method itself, rather than significant changes in manufacturing or bioperformance of the product. Even when a dissolution method is properly developed, without documenting critical parameters such as sampling procedure, dissolution results can be sensitive to testing conditions, potentially compromising their ability to provide insight into quality changes.

Upon careful review of the commentary’s content, it was noted that some statements regarding the study on potential variability caused by sampling cannula size and setting were inconsistent, vague, or inaccurate. It would be beneficial to address these inaccuracies concerning the importance of defining and documenting both sampling cannula size and sampling procedure settings. We remain open to further engagement on this topic to ensure clarity and accuracy in dissolution testing methodologies.

## Inconsistent Statements in the Commentary

The commentary asserts that efforts should be made to minimize the impact of sampling procedures and probes on dissolution results. We concur with this statement, as it aligns with our published findings. Our research indicates that “utilizing sampling cannulas with an outer diameter (OD) less than 3.6 mm may be necessary to minimize errors in dissolution results, particularly for drug products sensitive to medium hydrodynamics.” Our data demonstrates that “disturbances can be minimized by employing sampling cannulas with an OD less than 3.6 mm and eliminated with an OD less than 1.7 mm, regardless of cannula setting.”

Based on our study, we concluded that “the size of the sampling cannula significantly influences dissolution results, and specifying the use of small OD sampling cannulas (less than 3.6 mm) during the sampling procedure may be necessary.” However, the authors from the IQ’s DWG express disagreement with this recommendation, stating that “restricting the use of sampling cannulas to those having an outer diameter less than 3.6 mm is unnecessary.” It is worth noting that our recommendation aligns with their suggestion to minimize negative effects through the use of smaller sampling cannulas.

## Unclear Statements in the Commentary

The commentary on our study contains several statements that are not directly related to our published results, conclusions, and recommendations. This response aims to clarify potential misunderstandings and address relevant points.

The commentary mentioned multiple times that “… none of the pharmacopeia considers the sampling device to be part of the dissolution assembly.” and “… the qualification status of a dissolution apparatus is not and should not be linked to the sampling procedure.”

Our study did not consider the sampling device as part of the dissolution assembly, nor did we link the qualification status of a dissolution apparatus to the sampling procedure. We acknowledged that the sampling procedure is separate from apparatus qualification by stating, “the sampling cannula is potentially one of the essential testing factors that can contribute to variability in dissolution testing, even for a calibrated dissolution system.” Our recommendation to include the size of the sample cannula and the type of sampling procedure in controlled quality documents is based on our findings that these parameters may introduce significant variation, even when the dissolution assembly meets USP requirements.

Regarding the validation of sampling procedures, we cited USP general chapter < 1092 > in our introduction, which states that sampling probes or fiber-optic probes can disturb vessel hydrodynamics, necessitating adequate validation. We also noted that current USP recommendations lack guidance on the outer diameter of sampling probes/cannulas and their placement during testing.

The commentary emphasizes the importance of validating and evaluating sampling procedures, which aligns with our study’s objectives. However, it is also important to note that the objective of our study “was to investigate whether various size and setting of sampling cannula yield different dissolution results using USP apparatus 2”.

The commentary’s conclusion that “The types of issues described in the paper relating to cannula and sampling procedures are unlikely to occur if dissolution scientists follow the existing guidelines in dissolution method development and validation and perform adequate robustness and ruggedness studies” may be unclear and subject to misinter-pretation. Our findings suggest that even when dissolution scientists adhere to existing guidelines and conduct thorough method development, validation, and robustness studies, analytical errors may still occur during method transfer if details of cannula and sampling procedures are not clearly documented and replicated.

## Inaccurate Statements in the Commentary

IQ’s DWG is concerned that “the conclusions published in the publication could raise expectations that experimental conditions, which are described in the dissolution procedure, must specify cannula size and sampling procedure even when the dissolution results are not affected by these”. This concern appears to be based on a misinterpretation of our research objectives and findings. Our study aimed to evaluate the impact of various sampling cannula sizes and settings on dissolution results using the USP 2 apparatus. The results indicated that both the size and setting of the sampling cannula may introduce significant systematic errors, even in calibrated dissolution apparatus. Consequently, inclusion of sampling procedure details is intended to mitigate potential analytical errors during method transfer and verification by third parties. Even if dissolution results are confirmed not to be affected by the sampling procedure during method development, documenting this information in controlled quality documents does not impose an undue burden on developers.

Regarding the phenomenon of coning, it is well-established that prednisone tablets disintegrate into particles during dissolution testing, forming a cone-shaped accumulation at the vessel bottom. Prednisone disintegrating tablets have a long history of use in studying variability sources associated with compendial USP dissolution apparatuses (3–9). The commentary acknowledges that prednisone disintegrating tablets are sensitive to fluid hydrodynamics, which aligns with our study’s objectives to identify fluid hydrodynamic changes caused by different sample cannula sizes. However, the commentary inaccurately claims that “(coning) causing inconsistent results between different tests on the same or similar material. This makes the comparison and analysis of the results difficult. Therefore, the results presented in the paper showing sensitivity to minor hydrodynamic changes are unsurprising and hold little relevance to a robust and well-developed dissolution procedure.” Notably, the U.S. Pharmacopeia (USP) utilizes similar prednisone disintegrating tablets as reference standard material for performance verification tests to assess proper dissolution apparatus performance (10–12).

Our study focused on the effect of sample cannula on dissolution results. The sensitivity of the test sample to detect variability caused by different cannula size and setting serves as an example of the effect for one drug (prednisone) and may be impactful for testing of other drug products with similar sensitivity to cannula size and setting, regardless of whether 50% or 100% release occurs during the testing period. Our multiple tests with prednisone disintegrating tablets under various conditions consistently demonstrated a clear effect of different sample cannula sizes on dissolution results. These findings illustrate that such information on test conditions that impact results are needed for ensuring robust dissolution testing procedures.

The commentary’s suggestion to avoid coning when developing a dissolution procedure during formulation development is not directly relevant to our study, which our study was focused on potential variability in dissolution methods. For our purposes, the prednisone disintegrating tablet was an appropriate choice due to its sensitivity to hydrodynamic changes.

Regarding automated sampling, our study utilized an automated sampling system (AutoMag) to get dissolution results with and without tip filters. We acknowledge that automated sampling is common practice. Our recommendation to avoid sampling procedures requiring filters at the end of each sampling cannula is based on the observation that tip filters can significantly increase the outer diameter of sampling cannulas, potentially causing large variability.

We recognize that some automated sampling systems require tip filters to prevent post-sampling dissolution from undissolved particles. However, advancements in instrument design have led to various filtration methods, including out-of-vessel filtering and particulate rinsing. Several commercially available dissolution testers offer automated sampling assemblies with filtration outside the vessel, demonstrating that our recommendations should not limit the deployment of automated dissolution instruments.

## Conclusion

Understanding potential sources of variability is necessary for ensuring reliable results when utilizing standardized systems for *in vitro* dissolution testing. While not part of the harmonized dissolution assembly, sampling cannulas are commonly employed to withdraw sample aliquots from the dissolution medium. The sampling cannula and associated procedures may be factors that contribute to variability in dissolution testing results.

Although studies from the 1980s demonstrated potential impacts of large-diameter sampling cannulas on dissolution results, and the United States Pharmacopeia (USP) recommended adequate validation, current practices for selecting sampling cannulas for dissolution testing often lack rigorous considerations and necessary documentation.

The use of smaller sampling cannulas (outer diameter < 3.6 mm) may be necessary to minimize errors in dissolution results, particularly for drug products sensitive to medium hydrodynamics. It is advisable to avoid the current practice of attaching filter tips to sampling cannulas, as this may significantly increase the outer diameter.

Intermittent sampling cannula setting may introduce greater variability in dissolution results compared to stationary setting. Current recommendations that advocate for intermittent sampling cannula setting to mitigate hydrodynamic disturbances may warrant reconsideration.

Issues related to sampling cannulas and procedures are particularly likely to arise during method transfer or verification by third parties when sampling information is incomplete or absent. The International Pharmaceutical Quality (IQ) Consortium’s Dissolution Working Group recommends that “sampling technique and cannula size should be specified in the dissolution procedure, which is part of the product specification, and should not be included in standard operating procedures (SOPs).” While acknowledging potential differences in quality document requirements, it is acceptable for details on sampling cannulas and procedures to be documented in controlled quality documents. We also acknowledge that further studies with a wider range of drugs would be beneficial to establish the generalizability of our findings.
